# Quantitative assessment of synergistic effects in push-pull tactical ventilation during nonlinear space fires

**DOI:** 10.1371/journal.pone.0353911

**Published:** 2026-07-24

**Authors:** Pei Wang, Yanbo Yin, Wenju Liu, Gang Wang, Yunchao Bai, Changyuan He

**Affiliations:** 1 School of Technology for Sustainability, Beijing Normal University, Zhuhai, China; 2 China Fire and Rescue Institute, Beijing, China; 3 Department of Airport Management, Civil Aviation Management Institute of China, Beijing, China; 4 Global Talent Development Center, Civil Aviation Management Institute of China, Beijing, China; University of Salerno: Universita degli Studi di Salerno, ITALY

## Abstract

Smoke generated in compartment fires rapidly degrades visibility and tenability, making mechanical tactical ventilation a critical measure for restoring operable conditions. This study provides a full-scale experimental assessment of the mechanical (fan-driven) component of tactical ventilation in a two-story, non-linear layout with stairwell connectivity. Following the cold-flow experimental methodology established in prior PPV studies, smoke was produced using commercially available simulated smoke cakes to generate cold, thereby isolating the aerodynamic ventilation performance from buoyancy-driven fire dynamics. The performance of positive pressure ventilation (PPV), negative pressure ventilation (NPV), and their coordinated push-pull operation was quantified using illuminance recovery, local airspeed measurements at openings, and clearance time to 90% illuminance recovery. Each condition was repeated three times. Results show that the push-pull mode achieved the shortest mean clearance time (101.4 ± 16.9 s, mean ± SD, n = 6), reducing clearance time by approximately 25.0% compared with PPV (135.1 ± 15.9 s) and 31.9% compared with NPV (149.0 ± 15.9 s). One-way ANOVA on the overall clearance time confirmed statistically significant differences among the three conditions (F(2,15) = 13.67, p < 0.001). However, the synergy gain factor (SGF = 0.666) indicates sub-linear gains relative to ideal linear superposition. Importantly, the findings are intended as a ventilation-driven baseline relevant to training scenarios and post-knockdown smoke management where thermal driving is reduced; extrapolation to fully developed fires requires coupled thermal-fluid validation.

## 1. Introduction

Smoke produced during building fires is a primary cause of casualties and a major obstacle to firefighting operations, as high-temperature toxic gases reduce tenability, impair visibility, and complicate rescue efforts [1–3]. Tactical ventilation, which establishes controlled airflow paths through mechanical means, is therefore critical to improve interior conditions and enhance firefighting effectiveness [4]. Among available methods, positive pressure ventilation (PPV) and negative pressure ventilation (NPV) are widely applied. When combined, they form a push-pull system that is theoretically expected to accelerate smoke removal by coordinating supply and exhaust, yet its actual performance in realistic building and aircraft geometries remains poorly quantified.

In recent years, extensive researches have examined building fire smoke, addressing its generation, propagation dynamics, and suppression through mechanical ventilation [5,6]. Previous research has primarily focused on simplified or linear geometries such as tunnels and corridors, where airflow paths are relatively straightforward. In these environments, the effects of multiple fans can often be approximated by linear superposition, and the synergistic gains can be commonly observed [7]. For example, Yan. and Iya et al. [8,9] conducted Computational Fluid Dynamics (CFD) simulations of mechanically ventilated multi-compartment fires, reporting reasonable agreement between numerical predictions and experiments for flow rates and pressure distributions. Cao et al. [10] further examined the influence of fire strength and ventilation openings in compartments, confirming the sensitivity of smoke transport to boundary conditions. Studies on tunnel fires also provided valuable insights. Hu et al. [11] evaluated smoke exhaust efficiency in scaled tunnel models, demonstrating that multiple exhaust points can improve efficiency. These works collectively highlight how ventilation performance is strongly influenced by configuration, fire intensity, and geometric boundary conditions.

Beyond fire scenarios, push-pull ventilation has been extensively studied in occupational environments. Zhao et al. [12] proposed a push-relay-pull system for multi-room buildings, showing that coordinated supply and exhaust can significantly improve age of air and reduce infection risk. Auerswald et al. [13] examined push-pull devices in residential chambers, finding that volumetric flow rates alone do not adequately describe air exchange effectiveness. These findings emphasize the promise of push-pull concepts in enhancing ventilation efficiency.

Recent studies have also highlighted the role of complex geometries and external conditions. Li et al. [14] demonstrates that using two air supply outlets near the fire floor is a more efficient pressurization method for stairwell smoke control than multiple distributed outlets in high-rise buildings. Hao et al. [15] modeled smoke spread in mine ventilation networks with multiple branches, showing that geometry and scale greatly influence diffusion and evacuation conditions. Węgrzyński et al. [16] demonstrated that external wind can substantially alter smoke venting in large warehouse fires, sometimes degrading exhaust performance. Zhou et al. [17] analyzed obstacle effects on push-pull flow fields, confirming that flow separation and nonuniformities become pronounced in obstructed layouts. In fire engineering, researchers have increasingly recognized the importance of nonlinear and complex building geometries [18,19].

Although these studies provide useful knowledge, several limitations remain. First, most fire ventilation research emphasizes linear or idealized spaces, whereas actual residential and office buildings are nonlinear with multiple compartments, stairs, and corners. Secondly, while push-pull systems have shown promise in occupational and ventilation science, their performance in realistic fireground tactical operations has rarely been quantified.

Based on these gaps, this study aims to conduct full-scale experiments to evaluate PPV, NPV, and push-pull strategies in a representative nonlinear building layout. We hypothesize that the push-pull strategy achieves superior smoke removal efficiency compared to single-mode PPV or NPV, but that its collaborative effect will fall short of ideal linear superposition, resulting in SGF < 1. To verify this hypothesis, smoke clearance time, concentration decay, and airflow distribution are quantitatively measured and analyzed. The findings are expected to provide scientific evidence on the real performance of multi-fan collaborative ventilation in nonlinear spaces, deepen the understanding of underlying fluid dynamics, and offer practical guidance for improving tactical ventilation strategies in actual firefighting operations. It is worth noting that the nonlinear, compartmented layout investigated in this study also bears structural resemblance to aircraft cabin and cargo hold configurations, where angular turns, narrow passages, and multi-compartment connectivity similarly constrain ventilation flow paths. The methodological framework and performance indices developed herein may therefore inform future investigations of tactical ventilation in aircraft fire scenarios.

From a practical standpoint, current fireground guidance emphasizes coordinated ventilation and suppression based on fire dynamics and situational risk assessment (e.g., NFPA 1700). Engineering smoke management design guidance is also available through standards such as NFPA 204, while fire service research programs (e.g., UL FSRI and NIST) have highlighted the sensitivity of ventilation outcomes to timing, flow paths, and compartment connectivity. These frameworks motivate the need for quantitative, full-scale data in geometrically complex enclosures. In this context, the present study focuses on the ventilation-driven baseline (with minimized thermal driving) to quantify how non-linear geometry constrains multi-fan synergy.

## 2. Methods and experimental setup

**Fig 1:**
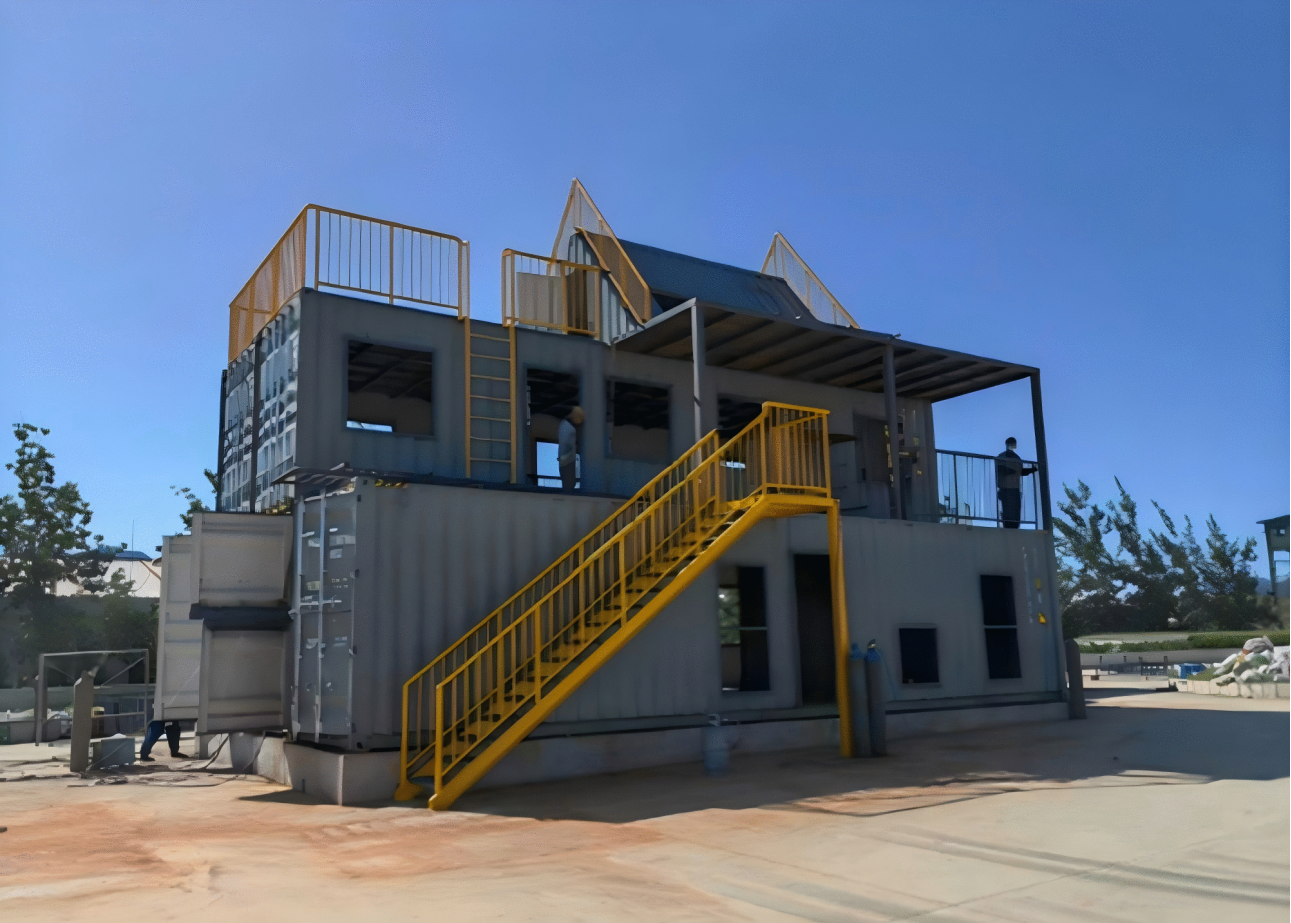
Schematic of the full-scale experimental facility (Photograph taken by the authors at China Fire and Rescue Institute).

The experimental design of this study follows the principles of reproducibility and precision, relying on a full-scale experimental facility, as illustrated in Fig 1.

The full-scale experimental site is in a container-based real fire training simulation facility, which is divided into two floors and can be used to simulate fire scenes in civil buildings, aircraft cabins, etc., for full-scale fire experiments and real fire training for firefighters.

### 2.1. Experimental Principle

Smoke concentration is quantified using a photometric method based on the Beer-Lambert law:


$$\begin{array}{c} I=I_{\text{0}}e^{-KL} \end{array}$$ (1)

where *I* is the transmitted light intensity after passing through smoke, $I_{\text{0}}$is the incident light intensity, *K* is the extinction coefficient (proportional to smoke concentration), and *L* is the optical path length. According to the law, the attenuation of light depends on both the extinction coefficient *K* and the path length *L*. By measuring the ratio of transmitted to incident intensity, the smoke concentration can be indirectly determined. Furthermore, the recovery rate of illuminance is employed to characterize smoke removal efficiency. A smoke-clearing efficiency index is defined as:


$$\begin{array}{c} R=\frac{I_{t}-I_{s}}{I_{\text{0}}-I_{s}}\times \frac{\text{1}\text{0}\text{0}\%}{t} \end{array}$$ (2)

where R is the illuminance recovery rate, $I_{t}$ is the illuminance at time *t*, $I_{s}$ is the minimum illuminance at smoke-filled condition, and $I_{\text{0}}$ is the initial ambient illuminance.

### 2.2. Full-Scale Experimental Platform

**Fig 2:**
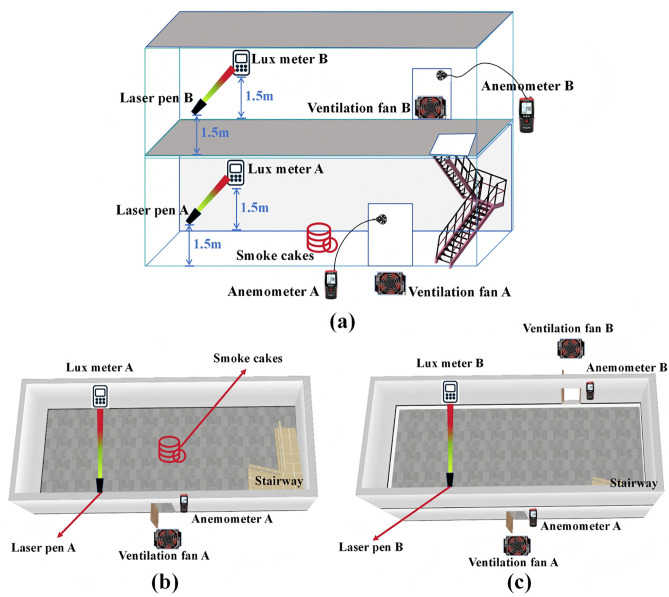
Schematic diagram of the experimental facility: (a) Overall view of the experimental scene; (b) Floor plan of the first floor; (c) Floor plan of the second floor.

The facility replicates a typical two-story residential layout, with a spiral staircase connecting the two floors. The detailed layout is shown in Fig 2, and the key parameters are summarized in Table 1.

**Table 1:** Key experimental parameters and conditions.

Category	Type/Specification	Value
Building model	Single floor size	12.19 m (L) × 4.87 m (W) × 2.90 m (H)
	Doorway opening	1.2 m (W) × 2.1 m (H)
Ventilation fan	Type	Mobile PPV fan (Honda GX160 based)
	Rated flow rate	19,810 m³/h
Instruments	Smoke source	Smoke cakes
	Anemometer	AS-806
	Lux meter	DELIXI DLY-1801C
	Light source	LED-500 laser pen
Environment	Ambient temperature	24 °C
	Ambient wind speed	< 0.7 m/s

Three ventilation conditions were tested for direct comparison:

Condition 1 (PPV): One fan placed 1.5 m outside the first-floor entrance (location A), blowing air inward.

Condition 2 (NPV): One fan positioned to extract smoke outward under negative pressure.

Condition 3 (Push-Pull): Two fans simultaneously deployed at the PPV and NPV positions.

All equipment was installed and calibrated prior to testing. Laser sources and lux meter probes were fixed at 1.5 m height in both first- and second-floor living rooms to monitor smoke concentration, and their response stability was confirmed in clear and smoke-filled conditions. Hot-wire anemometers were installed at 0.5 m, 1.0 m, and 1.5 m from the entrances on both floors, 0.4 m from the door frame, ensuring no interference with door operation. For each trial, 15 smoke cakes were ignited in a fire pan located at the center of the first-floor living room. Pre-experiments were conducted to verify data acquisition stability and overall system reliability. All instruments were checked for functional consistency before each test. The anemometer accuracy follows the manufacturer specification for AS806 (±3%rdg ± 0.1 m/s), and cross-checked under stable ambient lighting to ensure consistent relative recovery measurements.

### 2.3. Measurement parameters and procedure

Cold smoke with near-neutral buoyancy was deliberately selected as the tracer medium for this study. This methodological choice serves three specific objectives. First, it isolates the mechanical ventilation component from buoyancy-driven flow dynamics, enabling a controlled assessment of fan-driven clearance performance without the confounding effects of thermal stratification and plume entrainment. Second, it ensures high repeatability across trials, as cold smoke generation produces consistent optical attenuation properties, whereas real fire scenarios introduce significant variability in heat release rate, smoke yield, and combustion products. Third, it provides results directly applicable to post-knockdown smoke management and fire service training scenarios, where thermal driving is substantially reduced following fire suppression and the primary operational challenge shifts to residual smoke clearance through mechanical ventilation [4].

This cold-flow approach is consistent with the experimental methodology established in the tactical ventilation literature, where prior full-scale studies have characterized PPV performance under controlled cold-flow conditions to isolate fan-driven aerodynamics from variable thermal effects [20]. Within this established framework, the present results should be interpreted as a fan-driven aerodynamic baseline; the extension to thermally driven fire conditions requires future validation, as discussed in Section 4.4.

The standardized procedure was as follows: (1) Seal the facility and release smoke until a stable and uniform concentration was reached (verified when laser transmittance dropped to zero). Prior to fan activation, uniform smoke distribution on both floors was confirmed by monitoring illuminance sensor readings; fan operation was initiated only when the illuminance on both floors had dropped to near-zero values (< 50 lux), indicating comparable initial smoke concentrations across both levels. (2) Start the fans according to the designated experimental mode and open the ventilation openings. (3) Continuously monitor airflow distribution at the second-floor exit using anemometer arrays, and record illuminance recovery. (4) Smoke clearance was considered complete when illuminance recovered to 90% of its initial value. Illuminance was sampled at 10-s intervals throughout each trial. The 90% recovery time for each trial was determined by linear interpolation between the two adjacent sampling points bracketing the target value. Complete time-resolved measurements are provided in S1 Data.

### 2.4. Quantitative evaluation model

To comprehensively evaluate collaborative smoke exhaust efficiency, a quantitative model incorporating three indices was developed.

Absolute Performance Index (API): Represents absolute smoke-clearing speed, defined as the reciprocal of average recovery time:


$$\begin{array}{c} API\;=\frac{\text{1}}{T_{avg}} \end{array}$$ (3)

where $T_{avg}$ is the average smoke clearance time (s).

Fan Utilization Index (FUI): Measures per-fan efficiency, defined as the ratio of API to the number of fans:


$$\begin{array}{c} FUI\;=\frac{API}{N} \end{array}$$ (4)

where *N* is the number of fans used. The FUI quantifies clearance performance normalized by fan count rather than energy consumption in the thermodynamic sense, as actual power consumption was not measured in this study.

Synergy Gain Factor (SGF): Evaluates whether multi-fan performance exceeds, matches, or falls short of ideal linear superposition. Using the API of Experiment 1 (single PPV fan) as the baseline ${API}_{ref}$, the SGF is calculated as:


$$\begin{array}{c} SGF\;=\frac{{API}_{multi}}{N\times {API}_{ref}} \end{array}$$ (5)

where ${API}_{multi}$ is the measured performance of the multi-fan scheme. An SGF > 1 indicates enhanced synergy (“1+1 > 2”), SGF = 1 indicates neutral synergy (ideal linear superposition), and SGF < 1 reflects sublinear combined effect with efficiency loss.

### 2.5. Repeatability and uncertainty

Each ventilation condition was repeated three times (n = 3) under identical initial conditions, including ambient temperature, wind speed, smoke charge quantity, and sealing configuration. Reported clearance times and derived indices represent the mean ± standard deviation (SD) across the three trials. To evaluate the statistical significance of differences among the three ventilation conditions (PPV, NPV, and Push-Pull), one-way analysis of variance (ANOVA) was performed for each response variable separately (i.e., independent ANOVAs for 1F Recovery, 2F Recovery, and the Overall clearance metric). The “Overall” clearance time is calculated as a derived, aggregated metric to represent macroscopic building clearance and is not treated as an independent spatial observation alongside the floor-level data. When the ANOVA yielded a significant result (p < 0.05), Bonferroni-corrected post-hoc pairwise comparisons were conducted to identify which specific pairs of conditions differed significantly. All statistical analyses were performed using Python (SciPy v1.11).

### 2.6. Boundary condition specification

To ensure reproducibility, the detailed boundary conditions for each ventilation configuration are specified as follows. In Condition 1 (PPV), the fan was positioned 1.5 m from the first-floor entrance at an inclination angle of approximately 15° from horizontal, directed toward the center of the doorway. The first-floor entrance door and second-floor exit window (1.2 m × 1.0 m) were fully open; all other openings were sealed with aluminum tape to minimize uncontrolled leakage. In Condition 2 (NPV), the extraction fan was placed at the second-floor exit window with the same sealing protocol. In Condition 3 (Push–Pull), both fans were deployed simultaneously at their respective positions. The fan model (Honda GX160-based, rated flow 19,810 m^3^/h at 3,600 rpm) was operated at maximum throttle in all conditions.

## 3. Results

The experimental results are presented and analyzed in terms of illuminance recovery, airflow velocity distribution, clearance time, and synergistic efficiency. For clarity, Condition 1 corresponds to PPV, Condition 2 to NPV, and Condition 3 to the Push-Pull configuration.

### 3.1. Airflow velocities and momentum transfer

The airflow velocity measurements summarized in Table 2 reveal the aerodynamic basis for the observed optical recovery behavior. The Comprehensive Wind Speed Index, defined as the product of the average inlet and outlet velocities, quantifies the coupling strength between the inflow and outflow momentum.

**Table 2:** Comparison of wind speed data at entrances and exits for three schemes.

Condition	Average Inlet Wind Speed (m/s)	Average Outlet Wind Speed (m/s)	Comprehensive Wind Speed Index
PPV (Cond. 1)	5.3	3.2	17.0
NPV (Cond. 2)	0.5	5.8	2.9
Push–Pull (Cond. 3)	5.2	5.5	28.6

Condition 3 achieved the highest Comprehensive Wind Speed Index (28.6), approximately 4 times higher than PPV and NPV. This balanced momentum coupling produced a coherent pressure gradient and minimized recirculation zones, leading to a stable and directed flow channel across the structure.

In PPV, the strong inlet jet dissipated rapidly inside the compartment, reducing effective throughput and generating lateral vortices. Conversely, NPV created strong outlet suction but lacked sufficient inlet replenishment, producing diffuse and energy-inefficient flow. Both single-fan configurations therefore exhibited incomplete momentum continuity. The Push-Pull mode, by contrast, formed a stable, bidirectional momentum chain, efficiently transferring kinetic energy through the interior volume. This physical coherence directly correlates with the rapid illuminance recovery and superior clearance performance observed experimentally.

### 3.2. Illuminance recovery curves and flow dynamics

**Fig 3:**
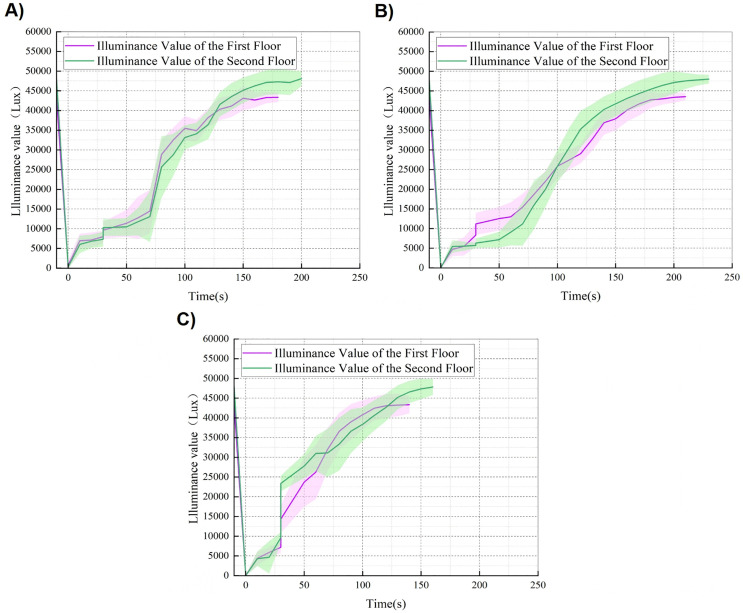
Recovery of illuminance values on each floor under the three ventilation conditions: (A) Condition 1 (PPV); (B) Condition 2 (NPV); (C) Condition 3 (Push–Pull).

**Fig 4:**
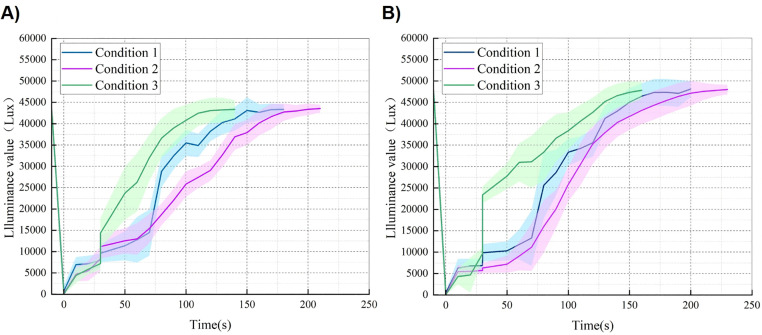
Recovery of illuminance under all three ventilation conditions, compared by floor: (A) first floor; (B) second floor.

Each condition was repeated three times (n = 3) under identical initial conditions; all reported values represent mean ± SD. Figs 3 and 4 include error bars representing the standard deviation across the three trials.

Under Condition 1 (PPV), the first-floor illuminance exhibited an immediate and steep increase following fan activation, reflecting a rapid local reduction in smoke concentration near the inlet. In contrast, the second-floor curve displayed a pronounced delay and a gentler slope, indicating that the induced airflow primarily displaced smoke within the lower compartment before gradually propagating upward. This staged and stratified recovery pattern demonstrates that the PPV mode generated a dominant localized jet rather than a continuous through-building airflow, resulting in delayed clearance of the upper floor.

Under Condition 2 (NPV), illuminance recovery on both floors was comparatively slow and exhibited an extended low-gradient phase, indicating limited bulk smoke displacement under single-fan extraction. Although outlet suction was strong near the exhaust, the overall clearing process was constrained by insufficient momentum supply and by flow path losses through the stair-connected compartments, leading to a prolonged clearance duration.

In contrast, Condition 3 (Push-Pull) exhibited the most rapid and synchronized illuminance recovery. The two floor curves showed minimal temporal lag and steep recovery gradients, suggesting that coordinated supply and exhaust established a more continuous through-compartment flow path. This behavior is consistent with improved smoke transport efficiency in stair-connected, non-linear enclosures, although the detailed internal flow structures require further validation (e.g., CFD or flow visualization).

Fig 4(A) further highlights the comparative performance on the first floor. The Push-Pull condition achieved the steepest slope and the fastest approach to baseline illumination, followed by PPV. The contrasting recovery gradients illustrate two fundamentally different clearance dynamics: localized and temporally segmented under single-fan operation versus globally synchronized and momentum-coupled under the Push-Pull strategy. This distinction illustrates the improved clearance performance when bidirectional flow fields are established within multi-level confined spaces [21].

Fig 4 present the temporal evolution of illuminance recovery on the first and second floors under all tested conditions, revealing the intrinsic dynamics of smoke clearance across vertically connected compartments. On the first floor, the Push-Pull condition exhibited the steepest slope and the fastest return to baseline illumination, followed by PPV and then NPV. This indicates that the dual‐fan configuration not only enhanced the total momentum input but also established a coupled pressure field that enabled smoke removal to proceed almost synchronously across both floors [22]. In contrast, the single‐fan modes demonstrated more segmented and localized recovery behavior, with illumination improvement occurring in discrete stages as the flow field evolved.

On the second floor, the advantages of the Push-Pull condition were even more pronounced. The illumination curve exhibited a continuous and monotonic recovery trend, suggesting that the upward propagation of smoke was effectively suppressed by the bidirectional airflow. Under the PPV condition, although the illuminance eventually recovered, the process was notably delayed due to the insufficient penetration of supply air through stairwell-induced vortices and pressure losses. The NPV condition performed worst, showing a prolonged plateau period and significant temporal lag, implying backflow and uneven smoke stratification in upper zones.

These comparisons illustrate that the Push–Pull ventilation strategy achieves improved smoke-clearing performance through combined fan operation. The synchronized clearance observed in both figures demonstrates that the bidirectional pressure coupling restructures the internal flow field.

It is noted that although the overall 90% illuminance recovery times for Conditions 1 and 2 were comparable (Table 3), the spatial and temporal profiles of recovery differed substantially, as shown in Fig 4. This apparent discrepancy arises because the recovery time metric captures only the endpoint of clearance, whereas the illuminance curves reveal differences in the intermediate clearance dynamics, including the rate and shape of recovery.

**Table 3:** Comparison of 90% illuminance recovery time (mean ± SD, n = 3 per floor per condition).

Condition	1F Recovery (s)	2F Recovery (s)
**Floor-Level Inferential Metrics**		
PPV (Cond. 1)	127.5 ± 14.7	142.8 ± 15.4
NPV (Cond. 2)	155.2 ± 16.7	142.8 ± 15.4
Push–Pull (Cond. 3)	88.9 ± 13.0	113.9 ± 8.5
ANOVA	F(2,6) = 14.97, p = 0.005**	F(2,6) = 4.58, p = 0.062
**Aggregated Descriptive Metric (Derived)**	**Overall Recovery (s)**	
PPV (Cond. 1)	135.1 ± 15.9	
NPV (Cond. 2)	149.0 ± 15.9	
Push–Pull (Cond. 3)	101.4 ± 16.9	
ANOVA	F(2,15) = 13.67, p < 0.001***	

### 3.3. Clearance time evaluation

The 90% illuminance recovery time was calculated for each of the three repeated trials under each condition. Table 3 presents the mean ± SD for each floor and overall. To prevent statistical ambiguity, the table is divided into primary floor-level measurements and the aggregated descriptive metric.

As clarified in the methodology, the “Overall” recovery time is a descriptive, derived metric aggregated from the facility-wide clearance profile, rather than an independent spatial observation. Separate one-way ANOVAs were conducted across the three ventilation conditions (PPV, NPV, Push-Pull) for each column individually to maintain statistical independence. A one-way ANOVA on the derived overall data revealed statistically significant differences in the macroscopic 90% illuminance recovery time among the three ventilation conditions (F(2,15) = 13.67, p < 0.001). Bonferroni-corrected post-hoc comparisons (Table 4) indicated that the push–pull strategy achieved significantly faster clearance than both NPV (mean difference = 47.6 s, p = 0.002) and PPV (mean difference = 33.8 s, p = 0.015). No significant difference was observed between PPV and NPV (p = 0.487). On the first floor, the ANOVA also yielded significant results (F(2,6) = 14.97, p = 0.005), with push–pull significantly outperforming both single-fan conditions. On the second floor, the differences did not reach significance (F(2,6) = 4.58, p = 0.062), likely attributable to higher variability associated with the nonlinear airflow path through the stairwell to the upper story.

**Table 4:** Post-hoc pairwise comparisons (Bonferroni-corrected, overall data).

Comparison	Mean Difference (s)	p-value (Bonferroni)	Significance
PPV vs NPV	13.8	0.487	n.s.
PPV vs Push–Pull	33.8	0.015	*
NPV vs Push–Pull	47.6	0.002	**

The push–pull strategy reduced the mean overall clearance time by 25.0% compared with PPV and 31.9% compared with NPV.Furthermore, the inter-floor time gap under push–pull was 25.0 s (1F: 88.9 s vs 2F: 113.9 s), confirming that the dual-fan configuration not only accelerates clearance but also moderately improves vertical uniformity.

The PPV mode demonstrated rapid initial recovery near the entrance but pronounced delay on the upper floor. NPV, while spatially more uniform, suffered from insufficient driving pressure and slow global kinetics.

### 3.4. Synergy and Efficiency Analysis

To provide a more intuitive comparison of ventilation performance, the Absolute Performance Index (API), Fan Utilization Index (FUI), and Synergy Gain Factor (SGF) were calculated based on the updated clearance time data from three repeated trials (Table 5).

**Table 5:** Comprehensive analysis of the synergistic smoke exhaust efficiency model.

Condition	N	T_avg (s)	API (s^−1^)	FUI (s^−1^)	SGF	p
PPV	1	135.1 ± 15.9	0.00740	0.00740	1.000	0.015*
NPV	1	149.0 ± 15.9	0.00671	0.00671	0.907	0.002**
Push–Pull	2	101.4 ± 16.9	0.00986	0.00493	0.666	—

Condition 3 (Push–Pull) achieved the highest API value of 0.00986 s^−1^, confirming its superior overall smoke clearance capability. However, its FUI was the lowest (0.00493), indicating that the additional fan did not yield proportionally higher per-fan performance. The SGF value of 0.666 quantitatively demonstrates that the dual-fan configuration produced a sublinear combined effect—roughly 33.4% lower than the idealized additive performance.

The observed results suggest that bidirectional coordination may induce an improvement in smoke exhaust performance, although the combined effect remains sublinear relative to ideal additive contribution.

## 4. Discussion

### 4.1. Mechanisms of sublinear synergy

The sublinear synergy gain factor (SGF = 0.666) provides direct experimental evidence that multi-fan ventilation in the nonlinear building layout examined here yields less-than-additive combined performance. While the magnitude of this sublinear effect is a measured outcome, the underlying flow physics were not directly resolved in the present experiments; we therefore propose two interrelated mechanisms that may plausibly account for it.

Boundary-induced energy dissipation. Unlike linear corridors, where multi-fan flows reinforce a unidirectional piston effect, the nonlinear geometry investigated here introduces pronounced flow-path tortuosity, so that the inflow jet delivered by the PPV fan encounters structural boundaries almost immediately. Prior work on forced-ventilation dynamics indicates that the interaction between a confined forced jet and bounding surfaces can incur substantial kinetic-energy losses [23]. In the context of this study, the spatial confinement restricts the free expansion of the stream, converting a portion of the forward kinetic energy into macroscopic boundary frictional losses and forcing abrupt changes in the bulk flow direction.

Furthermore, intermediate volumes such as stairwells complicate the continuous transfer of momentum between the supply and exhaust points. As demonstrated by Domingo et al. [24] in their analysis of complex underground transport interchanges, structural discontinuities and intermediate spaces often hinder optimal flow coupling, causing portions of the mechanical energy to dissipate without effectively driving bulk smoke removal. This structural hindrance disrupts the direct momentum transfer, leading to uneven kinetic energy distribution and delayed clearance in peripheral zones, as reflected in the second-floor illuminance curves. This macroscopic interpretation also aligns with findings in residential push-pull ventilation devices [25], confirming that architectural layout constraints inherently dictate systemic efficiency degradation, thereby explaining the sublinear synergy observed in our experiments.

Taken together, these observations support the interpretation that nonlinear architectural geometry tends to amplify dissipative losses even under mechanically driven ventilation, offering a plausible explanation for the sublinear synergy observed here. These mechanistic interpretations remain hypothetical, however, and warrant future validation through pressure-field measurements, flow visualization, or CFD simulation.

### 4.2. Comparison with Previous Studies

Prior studies of tunnel and corridor ventilation consistently reported super-additive or near-linear fan interactions, with SGF values often at or above unity [3,21]. In such linear spaces, collaborative fan use frequently yields a measurable gain, as unidirectional piston flows are reinforced rather than disrupted.

However, recent research in compartmentalized buildings suggests otherwise. Wirnsberger et al. [26] combined CFD simulations with full-scale apartment measurements and found that push-pull ventilation improved the overall “age of air” index but still left zones of stagnation in corridor-room junctions. This aligns with our finding that in nonlinear layouts, absolute performance improves with fan pairing, but marginal efficiency falls below ideal superposition.

Similarly, Ionescu et al. [27] showed through PyroSim simulations of multi-story structures that PPV improved visibility and thermal conditions compared to natural ventilation, but efficiency was constrained by vertical transport and smoke accumulation in stairwells. Our full-scale results corroborate this, demonstrating that nonlinear geometry imposes systemic efficiency losses even when absolute clearance is enhanced.

In addition, Liu et al. [28] employed large-eddy simulation to study PPV in multi-chamber cabin, reporting delayed clearance and smoke ingress despite mechanical pressurization. This finding parallels the delayed clearance observed in the second floor during our PPV experiment, highlighting the limitations of single-fan strategies in multi-level structures.

Together, these studies reinforce the divergence between linear infrastructure contexts—where fan synergy can be assumed beneficial—and nonlinear architectural contexts, where complex airflow interactions demand case-specific analysis and cautious tactical planning.

### 4.3. Operational Implications for Firefighting

The Push–Pull strategy remains most effective. Clearance times were shortened by 25% relative to PPV, and ANOVA confirmed that both push–pull vs PPV (p = 0.015) and push–pull vs NPV (p = 0.002) differences were statistically significant. The recommendations here are consistent with recent findings that fan placement, geometry-driven flow constraints, and local pressure differences significantly mediate ventilation outcomes [12,17].

Incident commanders should: (1) Deploy Push–Pull when two fans are available. (2) Avoid extrapolating tunnel-based findings to compartmentalized buildings. (3) Assess stairwell connectivity to minimize recirculation.

### 4.4. Limitations and Future Research Directions

#### 4.4.1. Cold smoke and thermal buoyancy.

The most significant limitation is the use of cold smoke, which eliminates thermal buoyancy, plume entrainment, and thermal stratification. In real fires, buoyancy creates a strong upward driving force and produces two-layer stratification [1,2]. However, this is a deliberate design choice following the variable isolation principle widely adopted in prior full-scale tactical ventilation studies [29,30]. Hot smoke would likely enhance clearance of all three strategies similarly; the relative ranking and sublinear SGF reflect geometric constraints independent of temperature. The cold-smoke results also directly apply to training exercises and post-knockdown smoke management [4].

#### 4.4.2. Dimensional analysis.

Results are in absolute terms. The nominal ACH was calculated: ≈ 57.6 h^−1^ for single-fan and ≈115.2 h^−1^ for push–pull (rated flow 19,810 m^3^/h, building volume ≈344 m^3^). Future studies should develop nondimensional scaling including Richardson number.

#### 4.4.3. Future research program.

We recommend a three-stage program: (1) hot-smoke experiments with controlled pool fires; (2) validated CFD (LES) calibrated against both datasets; (3) parametric studies varying fan capacity, placement, and building geometry.

## 5. Conclusions

This study conducted a systematic experimental evaluation of tactical ventilation in a nonlinear multistory structure. Each condition was tested three times. The results demonstrate that NPV provides uniform but slow clearance (149.0 ± 15.9 s), PPV achieves faster yet uneven clearance (135.1 ± 15.9 s), and Push–Pull combines speed with improved synchronization (101.4 ± 16.9 s). One-way ANOVA on the overall clearance time confirmed significant differences (F(2,15) = 13.67, p < 0.001), with push–pull significantly outperforming both PPV (p = 0.015) and NPV (p = 0.002).

Synergy analysis revealed a sublinear effect (SGF = 0.666). The cold-flow methodology, consistent with established practices, enables isolation of the aerodynamic component. Extension to fully developed fires requires hot-smoke experiments and CFD validation.

## Supporting information

S1 Data Raw experimental measurements. (XLSX)
